# Non-H3 CDR template selection in antibody modeling through machine learning

**DOI:** 10.7717/peerj.6179

**Published:** 2019-01-11

**Authors:** Xiyao Long, Jeliazko R. Jeliazkov, Jeffrey J. Gray

**Affiliations:** 1 Chemical and Biomolecular Engineering, Johns Hopkins University, Baltimore, MD, United States of America; 2 Program in Molecular Biophysics, Johns Hopkins University, Baltimore, MD, United States of America; 3 Sidney Kimmel Comprehensive Cancer Center, Johns Hopkins University, Baltimore, MD, United States of America; 4 Institute for Nanobiotechnology, Johns Hopkins University, Baltimore, MD, United States of America

**Keywords:** Protein structure, Structure prediction, Rosetta, Antibodies

## Abstract

Antibodies are proteins generated by the adaptive immune system to recognize and counteract a plethora of pathogens through specific binding. This adaptive binding is mediated by structural diversity in the six complementary determining region (CDR) loops (H1, H2, H3, L1, L2 and L3), which also makes accurate structural modeling of CDRs challenging. Both homology and *de novo* modeling approaches have been used; to date, the former has achieved greater accuracy for the non-H3 loops. The homology modeling of non-H3 CDRs is more accurate because non-H3 CDR loops of the same length and type can be grouped into a few structural clusters. Most antibody-modeling suites utilize homology modeling for the non-H3 CDRs, differing only in the alignment algorithm and how/if they utilize structural clusters. While RosettaAntibody and SAbPred do not explicitly assign query CDR sequences to clusters, two other approaches, PIGS and Kotai Antibody Builder, utilize sequence-based rules to assign CDR sequences to clusters. While the manually curated sequence rules can identify better structural templates, because their curation requires extensive literature search and human effort, they lag behind the deposition of new antibody structures and are infrequently updated. In this study, we propose a machine learning approach (Gradient Boosting Machine [GBM]) to learn the structural clusters of non-H3 CDRs from sequence alone. The GBM method simplifies feature selection and can easily integrate new data, compared to manual sequence rule curation. We compare the classification results using the GBM method to that of RosettaAntibody in a 3-repeat 10-fold cross-validation (CV) scheme on the cluster-annotated antibody database PyIgClassify and we observe an improvement in the classification accuracy of the concerned loops from 84.5% ± 0.24% to 88.16% ± 0.056%. The GBM models reduce the errors in specific cluster membership misclassifications when the involved clusters have relatively abundant data. Based on the factors identified, we suggest methods that can enrich structural classes with sparse data to further improve prediction accuracy in future studies.

## Introduction

Antibodies are central to adaptive immunity. They are responsible for recognizing a variety of target molecules known as antigens. They acquire the ability to recognize any one of a diverse set of targets through two biological mechanisms: V(D)J recombination and affinity maturation. These gene-editing mechanisms can produce an enormous quantity of unique sequences, in theory on the order of 10^13^ (Georgiou et al., 2014; DeKosky et al., 2016; Hou et al., 2016), though the antibody repertoire of any single individual comprises only a fraction of the possible sequences. Recent advances in high-throughput sequencing techniques are permitting unparalleled access to the human antibody repertoire (Boyd & Crowe, 2016; Luciani, 2016), thus furthering our comprehension of immune response to vaccination, infection, and autoimmunity. Beyond sequence data, structural information can provide additional insights about the functions of antibodies. Yet only a very small fraction of antibodies have solved crystal structures in the Protein DataBank, reported as 3,087 structures (Dunbar et al., 2014) with a filtered set of 1,940 PDB antibody entries included in PyIgClassify as of August, 2017 (Adolf-Bryfogle et al., 2015). Most of these structures are murine (51.15%) and human (35.51%), while repertoire sequencing is rapidly expanding our knowledge of other species. It would be challenging and time-consuming to close the gap between structure and sequence knowledge through experimental structure determination methods. Computational modeling provides a feasible alternative. For example, in chronic lymphocytic leukemia, models of antibody structures added prognostic value over sequence data alone (Marcatili et al., 2013). Besides using modeling to develop biological understanding, docking studies of antibodies complexed with various antigens can reveal atomic details of antibody–antigen interactions (Kuroda et al., 2012; Kilambi & Gray, 2017; Koivuniemi, Takkinen & Nevanen, 2017; Weitzner et al., 2017). Finally, in antibody design studies, computational approaches can enhance affinity or design an antibody de novo—without prior sequence information (Lippow, Wittrup & Tidor, 2007; Kuroda et al., 2012; Dunbar et al., 2016; Baran et al., 2017; Adolf-Bryfogle et al., 2018). To be useful, however, computational methods must be able to accurately predict antibody structure.

**Figure 1:**
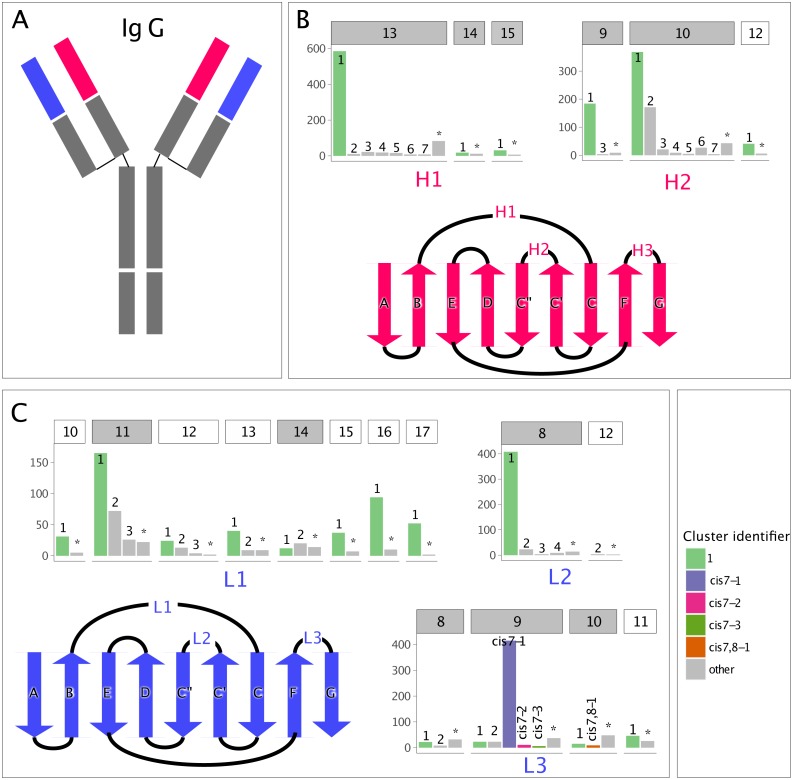
Clusters in canonical CDR loops are not balanced in their number of members. (A) IgG cartoon structure highlighting the variable heavy (VH, red) and light (VL, blue) domains, which bind antigen through their CDR loops. (B) Count of non-redundant CDR loops in the PyIgClassify database for each VH loop-length and -type cluster, with a gray header background indicating adequate numbers for GBM modeling and a white header background indicating inadequate numbers, and a cartoon highlighting the VH beta-strand connectivity and CDR loop location. The CDR H3 is excluded due to its highly variable nature. (C) Analogous to (B), but for the VL. The most populous cluster and clusters possessing cis-prolines are colored.

Typical approaches to antibody structure prediction decompose the problem into three parts based on known antibody-structural features (Almagro et al., 2014). Antibodies are typically comprised of a light and heavy chain, both having variable (V) and constant (C) regions (Fig. 1A). While the constant region is important for signaling, it does not vary across antibodies and does not greatly affect the antigen-binding function. On the other hand, the variable region can differ between antibodies and is responsible for recognizing antigens. The variable region can be further divided into a framework region (FR), with greek-key β-barrel topology, and six complementarity-determining regions (CDRs), which are solvent-exposed loops connecting the β-strands comprising the aforementioned β-barrel (Figs. 1B– 1C). The FR is conserved and has a low rate of mutation across antibodies, whereas the CDRs, and in particular the CDR H3, are highly mutable in order to be able to bind a wide variety of antigens (Schroeder, Cavacini & Cavacini, 2010). Thus, the antibody modeling problem is often decomposed into (1) homology modeling of light and heavy FRs, (2) homology modeling of the non-H3 CDR loops, and (3) *de novo* modeling of the CDR-H3 loop.

Of these three modeling problems, modeling the CDR-H3 loop is the most challenging. For example, an average backbone RMSD of 2.8  ±  0.4 Å  was reported over eleven test antibodies and seven modeling approaches in a recent blind assessment (Almagro et al., 2014). By comparison, FR modeling was found to achieve sub-angstrom accuracy, on average, for both the light and heavy chains. The quality of the modeling of the non-H3 CDRs was uneven, with average backbone RMSDs ranging from 0.5 ±  0.1 to 1.3 ±  1.1 Å for RosettaAntibody models of targets in the same assessment (Weitzner et al., 2014). This result was surprising since previous studies have found that, when divided by loop type and length (e.g., H1-10), non-H3 CDRs can be structurally clustered and a majority (85%) of the loops assume structures similar to just a few loops structures, called the cluster exemplars (North, Lehmann & Dunbrack, 2011). Whether antibody-modeling methods have been using this structural information effectively remains an open question.

In the four most popular methods SAbPred (Dunbar et al., 2016), PIGS (Marcatili et al., 2014), Kotai Antibody Builder (Yamashita et al., 2014) and RosettaAntibody (Weitzner et al., 2017), non-H3 CDR loops are generally modeled by homology: a CDR loop with a known structure is chosen as a template structure based on its sequence similarity to the query CDR loop. However, the use of additional structure-based rules, the scoring matrix used to determine sequence similarity, and the database of possible templates all vary among methods.

First, PIGS and Kotai Antibody Builder both use sequence-based rules to identify the structural cluster of the query CDR sequence. If a potential cluster or clusters can be identified, the methods constrain the template search to these clusters. While sequence rules are easy to interpret and can offer deterministic cluster assignments, they are limited in their adaptability and their power—as the number of known antibody structures and sequences grows, analysis by hand becomes more challenging. For example, the current PIGS method uses curated rules from a variety of previous studies (Marcatili et al., 2014). For the CDR H1 loop, it has four canonical clusters from four different loop lengths with sequence rules, but according to North, Lehmann & Dunbrack (2011) study there are now 17 structural clusters and six loop lengths for the CDR-H1 loop (North, Lehmann & Dunbrack, 2011). Another issue is that some clusters lack deterministic, human-identified rules. Kotai Antibody Builder (Yamashita et al., 2014) devised sequence rules for cluster identification in accordance with the clusters identified by North, Lehmann & Dunbrack (2011), but in that publication there are not clear sequence rules for distinguishing among H1 clusters. In fact, only a fraction of the remaining non-H3 CDR clusters (26/56) have sequence rules (Shirai et al., 2014) and, worryingly, not all sequence rules are comprehensive. For example, under the Chothia numbering convention (Chothia et al., 1989), an arginine at position 71 in length 10 CDR-H2 loops can indicate membership to either the H2-10-1 or H2-10-2 cluster, but not all sequences belonging to the H2-10 cluster have that arginine: only 8 out of 155 CDRs in H2-10-1 and 38 out of 42 CDRs in H2-10-2 do. To address this problem and the problem of inadequate sequence-based rule coverage, Kotai Antibody Builder built position-specific-substitution-matrix (PSSM) profiles for each cluster, so that when sequence rules fail, PSSM-based scoring can be used to suggest a cluster (Shirai et al., 2014). When assessed using the PyIgClassify antibody dataset, Kotai Antibody Builder correctly identified the cluster in 90% (Shirai et al., 2014) of all CDR loops, including the CDR H3. However, it is not clear whether the tested data was excluded from the construction of the PSSM profiles, so the reported accuracy might have been overestimated.

Recent assessments of antibody structural modeling report varying accuracy of non-H3 CDR modeling. When RosettaAntibody was benchmarked in a recent study on 54 antibody targets (Weitzner et al., 2014), non-H3 CDR loop modeling achieved sub-angstrom backbone RMSD between the homology-modeled and crystal-structure CDRs in 42/54 (L1), 50/54 (L2), 37/54 (L3), 36/54 (H1), and 42/54 (H2) cases. Meanwhile, using a set of 689 antibody structures and leave-one-out-cross-validation (LOOCV), PIGS (Marcatili et al., 2014) was found to capture ∼50% of the modeled non-H3 CDRs with sub-angstrom backbone RMSDs to the native CDRs structures. Finally, SAbPred (Dunbar et al., 2016) was tested on the same set of 54 antibodies as RosettaAntibody and resulted in average backbone RMSDs as 1.09, 0.59, 1.00, 0.88 and 0.90 Å for 5 non-H3 CDRs (Choi & Deane, 2011). Despite mostly sub-angstrom average RMSDs for all methods and benchmarks, individual models with RMSDs much greater than an angstrom were not rare (Choi & Deane, 2011; Weitzner et al., 2014; Almagro et al., 2014), suggesting a need for special handling of these fail-prone cases. We propose introducing an extra step to non-H3 CDR modeling, where a machine learning approach is used to predict cluster membership and template structures are only selected from the predicted cluster. We hope to improve accuracy by preventing templates coming from a structurally distinct cluster with a large structural distance to the query CDR loop.

Machine learning has been used extensively in protein classification problems. For example, machine learning based methods have accurately predicted protein function (Radivojac et al., 2013), folding rate (Corrales et al., 2015), super-family levels for fold recognition (Jain, Garibaldi & Hirst, 2009), enzyme classes (Kumar & Choudhary, 2012), and functional binding sites (Si, Zhao & Wu, 2015). For antibody structure, LYRA uses a similarity-score-based template selection method for modeling the antigen-binding site (Klausen et al., 2015). Decision-tree-based models have been used on antibodies to predict the structural classes of antigen binding regions. Chailyan et al. (2011) used a random forest model to predict non-H3 loop clusters with about 90% accuracy on the data set available then, which had ∼200 antibodies in 10 clusters, before the more complete North, Lehmann & Dunbrack (2011) clustering was developed. Messih et al. (2014) focused on the CDR-H3 loop and compared the template selection quality of a random forest model to the BLAST similarity score method on a dataset comprising of 401 structures. They found that, on average, the random forest model produced smaller between-query-template RMSD values. Today, more structures are available, and North, Lehmann & Dunbrack (2011) have provided a more comprehensive non-H3 CDR clustering scheme. Therefore, a new study using state-of-the-art machine learning prediction performance on canonical CDR loops is needed.

Of many machine learning methods, Gradient Boosting Machine (GBM) was recently shown to yield the best accuracy for structural classification of proteins in the Structural Classification Of Protein database (SCOP) (Jain, Garibaldi & Hirst, 2009). The GBM method builds a succession of a tunable number of weak learners, with each learner being a decision tree with tunable tree depth and branch splitting rules. During training, incorrectly classified samples are upweighted in later iterations to converge on final decision trees that fix errors. Since non-H3 CDR loop cluster prediction is a protein structural classification problem, we will adapt this approach.

In this work, we attempted to increase the quality of CDR structural template selection by using the machine learning method GBM. For a relevant and fair assessment, we evaluated the quality of template selection rather than that of the final model. The assessment was performed on the comprehensive dataset PyIgClassify (Adolf-Bryfogle et al., 2015), comparing the original RosettaAntibody structural template identification method and the GBM method developed herein. As the disparities of cluster member sizes can affect the performance of GBM (Sun et al., 2007), we surveyed various techniques for overcoming the data imbalance problem. Approaches vary, from down-sampling the majority class to up-sampling the minority classes, or even adding synthetic members to balance the size of the clusters (Chawla et al., 2002; Blagus & Lusa, 2013). Previous results suggest that the best approach depends on the specific data set and size (Kuhn & Johnson, 2013a). In our study, we used the up-sampling strategy.

We show that (1) the new GBM can better identify the query CDR’s structural cluster than RosettaAntibody and (2) selecting structural templates from within the query cluster results in lower RMSD templates than selecting outside the cluster. The GBM models also recapitulate previously known sequence motifs and identify new ones. The GBM models find that the presence or absence of a single residue on its own is not sufficient to assign a sequence to a specific structural cluster. Instead, the combination of residues in the query sequence is important for assigning a probable cluster. These findings suggest that incorporating machine learning methods may achieve closer-to-native templates selection during non-H3 CDR homology modeling and realize an automated feature selection, surpassing the manual curation of sequence rules.

## Materials and Methods

### Dataset

We compared the CDR structural class prediction performance of GBM and blindBLAST on the non-redundant CDR loops in the PyIgClassify database (http://dunbrack2.fccc.edu/PyIgClassify/Download/Download.aspx). The structures and clusters were downloaded in February 2017 by selecting the “CDRs and clusters of non-redundant sequences for a given CDR” database. The database contains antibody structures from the PDB with 2.8 Å or better resolution and an 0.3 R-factor cutoff, while excluding non-proline *cis* loops or loops with highly improbable conformations (North, Lehmann & Dunbrack, 2011). The set of non-redundant canonical CDR loops from the database is partitioned by CDR loop type and length. It contains 3,558 total loops from 1,153 distinct antibody structures.

In PyIgClassify, CDR loops are partitioned by type (e.g., L1 or L2) and length (e.g., 10 or 11) and clustered such that the members of each cluster are more structurally similar to their cluster exemplar than to the exemplar of any other cluster, with the exemplar as defined in North, Lehmann & Dunbrack (2011). The distribution of CDR cluster membership is unbalanced, with each CDR loop and length pair having one well-populated, dominant cluster and many sparsely populated, non-dominant clusters. In our study, CDR loops which were unable to find a nearest neighbor cluster within certain dihedral angle distance and clusters smaller than three members were merged into a single cluster labelled “none”. The cluster member size distribution by CDR loop and length type is shown in Fig. 1.

### Structural class prediction methods

We employed two methods, blindBLAST and GBM, for CDR structural class prediction. The blindBLAST approach comes from the current version of RosettaAntibody (Weitzner et al., 2017), which identifies template non-H3 CDR loops through a BLAST search against CDRs of the same length and type using the PAM30 matrix to rank sequence similarity. The BLAST parameters used are:

-substitution_matrix PAM30 -word_size 2 -max_target_seqs 3000 -evalue 2000

The template loop with the most sequence similarity to the query is then selected for grafting and further modeling. We refer to this approach as “blindBLAST”, as it does not utilize CDR structural cluster information but rather identifies the structural class of a CDR loop implicitly by choosing a template with the highest bitscore. On the other hand, we trained supervised GBM models for each CDR loop and length type. Each model learns to predict the structural class (synonymous to the structural cluster) from the labelled CDR sequences, including the 10 flanking residues on either side. Sequences were vectorized by one-hot-encoding (Beck & Woolf, 2000): the observed amino acid is represented by a one and the other possible 19 amino acids are zeros. Thus, a CDR loop of length 10 is represented by a 30*20 matrix.

We trained our GBM model by searching a hyper-parameter grid in a nested 3-repeat 10-fold CV scheme. As typical for nested CV, the grid search was performed in the inner loop (consisting of 3-repeat 10-fold CV on the training folds for each iteration of the outer loop), and model accuracy was assessed over the outer loop. We used CV instead of a single training/test data split to counter data sparsity. Fold splitting was stratified, ensuring that the composition of each fold was representative of the whole dataset (Kohavi, 1995)**.** To counter the unbalanced sample problem, classes with low population were up-sampled to either 50 or to the number of samples in the most popular cluster, whichever was lower (Dittman, Khoshgoftaar & Napolitano, 2015; Sun, Kamel & Wang, 2006). The hyper-parameters yielding the highest estimated model accuracy were used for the final model. All machine learning was performed using the Caret package (Kuhn, 2008).

### Comparisons of different methods

For both blindBLAST and GBM, an error case was identified when the query cluster did not match to the predicted (template) cluster. The number of error cases and the corresponding accuracy were calculated for each loop and length type for each repeat and then averaged over the three repeats. To further analyze failures, we counted and compared the specific misclassifications (i.e., the number times a cluster A to cluster B misclassification occurred) for both GBM and blindBLAST.

The *χ*^2^ goodness-of-fit test was run on each loop type and length combination to test whether the blindBLAST errors differed significantly from random assignment. The *χ*^2^ was calculated as

(1) \begin{eqnarray*}{\chi }^{2}=\sum _{\mathbf{Y }}\sum _{\mathbf{X}} \frac{({\epsilon }_{\text{blindBLAST}}^{\mathbf{X}\rightarrow \mathbf{Y }}-\mathbi{E}[{\epsilon }^{\mathbf{X}\rightarrow \mathbf{Y }}])^{2}}{\mathbi{E}[{\epsilon }^{\mathbf{X}\rightarrow \mathbf{Y }}]} ,\end{eqnarray*}

where ${\epsilon }_{\text{blindBLAST}}^{\mathrm{X}\rightarrow \mathrm{Y }}$ is the average error count of misclassifying cluster *X* to cluster *Y* in 3-repeats-10-fold cross-validation, $E \left[ {\epsilon }^{\mathrm{X}\rightarrow \mathrm{Y }} \right] ={n}_{\mathrm{X}}\cdot {P}_{\mathrm{Y }}$ is the expected error count of misclassifying cluster *X* to cluster *Y*, with *n*_X_ as the number of samples in cluster *X* in the dataset and *P*_Y_ as the fraction of cluster *Y* samples. The significance value *p*^X→Y^ corresponding to the *X* →*Y* misclassification is then found comparing to the *χ*^2^ distribution with degrees of freedom equal to the number of clusters. When *χ*^2^ exceeds the critical value, it means that blindBLAST is not different from random (*H*_0_ is rejected).

Additionally, raw error cases counts are confounded by the member size differences between structural classes, so we can’t compare the values directly. Instead, for a particular misclassification (e.g., H2-10-1 incorrectly classified as H2-10-4), we compare its blindBLAST error count to a simulated random error count distribution using a two-tailed hypothesis test at an 0.05 significance level (Eq. (1)). The random error counts are generated from 10,000 iterations of randomly assigning cluster identies in proportion to the naturally occurring rate. The comparison between the average blindBLAST error count over three repeats of 10-fold cross validation (${\epsilon }_{\text{blindBLAST}}^{X\rightarrow Y}$) and its simulated distribution (${\epsilon }_{n,\text{random}}^{X\rightarrow Y}$) hinges on the significance value, *p*, which is determined as the proportion of the random simulated error counts with smaller values than ${\epsilon }_{\text{blindBLAST}}^{X\rightarrow Y}$:

\begin{eqnarray*}{H}_{0}& :{\epsilon }_{\text{blindBLAST}}^{X\rightarrow Y} \text{is equivalent to} {\epsilon }_{\text{random}}^{X\rightarrow Y}, \end{eqnarray*}

(2) \begin{eqnarray*}p({\epsilon }_{\text{blindBLAST}}^{X\rightarrow Y})& = \frac{\sum _{n=1}^{10000}I \left( {\epsilon }_{\text{blindBLAST}}^{X\rightarrow Y},{\epsilon }_{n,\text{random}}^{X\rightarrow Y} \right) }{10000} ,\end{eqnarray*}

\begin{eqnarray*}\text{where} I \left( {\epsilon }_{\text{blindBLAST}}^{X\rightarrow Y},{\epsilon }_{n,\text{random}}^{X\rightarrow Y} \right) & = \left\{ \begin{array}{@{}l@{}} \displaystyle 1,\mathrm{if} {\epsilon }_{\text{blindBLAST}}^{X\rightarrow Y}\geq {\epsilon }_{n,\text{random}}^{X\rightarrow Y} \\ \displaystyle 0,\mathrm{if} {\epsilon }_{\text{blindBLAST}}^{X\rightarrow Y}\lt {\epsilon }_{n,\text{random}}^{X\rightarrow Y}  \end{array} \right. \end{eqnarray*}

We reject the null hypothesis, if *p* ≤ 0.025 (meaning blindBLAST misclassifies with significantly lower error than random) or if *p* ≥ 0.975 (meaning blindBLAST misclassifies with significantly higher error than random). Three categories of misclassifications are generated using the *p* values (Table S1).

### Structure and sequence metrics

To better understand why misclassification may have occurred, we computed two structural metrics and compared each of the metrics between the correct cases and the incorrectly predicted cases using the blindBLAST. Defining the dihedral angle distance by following (North, Lehmann & Dunbrack, 2011),

(3) \begin{eqnarray*}D \left( i,j \right) ={\mathop{\sum \nolimits }\nolimits }_{r=1}^{N}D \left( {\phi }_{r}^{i},{\phi }_{r}^{j} \right) +D \left( {\psi }_{r}^{i},{\psi }_{r}^{j} \right) ,\end{eqnarray*}

where $D \left( {\theta }_{1},{\theta }_{2} \right) =2(1-\cos ({\theta }_{1}-{\theta }_{2}))$, *N* is the length of the loop, *r* is the residue number, and *i* and *j* represent each CDR identity in the CDR pair for the dihedral angle distance calculation. First, we calculated the dihedral angle distance of every query case to the exemplar of its corresponding cluster and compared the distance distributions for correctly and incorrectly classified cases. Second, we counted the number of structural neighbors in the cognate cluster for all CDR loops. A structural neighbor is defined to be any CDR loop with dihedral angle distance to the query less than 1/15th of the radius of that cluster, where the radius is the largest dihedral distance between the cluster exemplar and any CDR loop in the cluster. We compared the distributions of the number of structural neighbors for the correctly and incorrectly classified cases. We also computed the dihedral angle distance between clusters that are significantly better distinguished by the blindBLAST than random by the *χ*^2^ test, and we compared the values to those of cluster pairs that are found to be nonsignificant in the *χ*^2^ test.

In addition to investigating structural features, we extracted sequence features from the tuned GBM models based on the scaled feature importance. Absolute importance was calculated by determining how much a decision tree split reduces Gini impurity (Louppe et al., 2013) and then summing over all node-size-weighed reductions on splits corresponding to that feature over all boosting trees (Kuhn & Johnson, 2013b). The importance was then scaled to values from 0 to 100.

The code for the model generation and analysis can be found in https://github.com/xlong2/machine-learning-cdr.

## Results

### BlindBLAST is more accurate than random assignment for all but one CDR loop type and length

**Figure 2:**
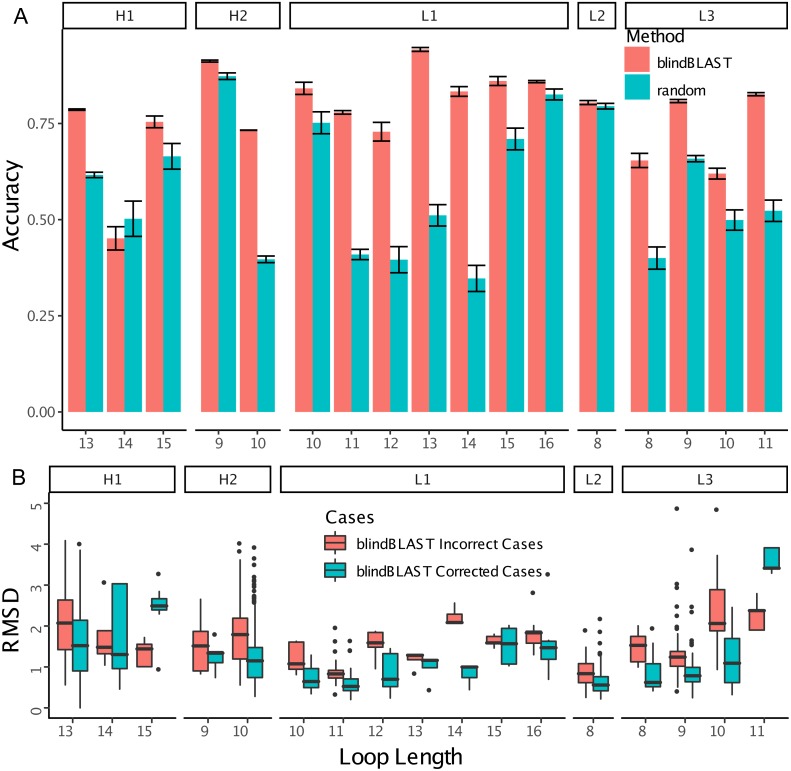
BlindBLAST can more accurately assign CDR loops than random, and, when assignment is to the cognate cluster, RMSD is lower. (A) Cluster assignment accuracy comparison between blindBLAST and random assignment. Error bars show the standard deviation of the accuracy. For blindBLAST, the standard deviation is calculated across the 3-repeat 10-fold cross validation, whereas for the random model it is the standard deviation of accuracies over 10,000 iterations. In all but one case (H1-14), blindBLAST determines clusters more accurately than random. (B) Comparison of query–template RMSD when the loop is selected by BLAST from the incorrect versus the “corrected” cluster. Incorrect cases are loops with templates from clusters other than the query and they are “corrected” by sequence alignment to only templates within their cluster. In most cases, BLAST finds lower-RMSD templates within the cognate loop cluster than outside of it, indicating that correct cluster determination from sequence can lead to a better structural template. However, two loop types (H1-15 and L3-11) do not have lower RMSD templates in their cognate cluster.

In blindBLAST, cluster assignment accuracies varied among the different CDR loop and length types from below 50% to almost 100% according to 3-repeat 10-fold cross-validation, as shown in Fig. 2A. In most of the cases where the clusters of the query and the template CDR did not match, a more near-native structural template could be found if the BLAST search was restricted to within the query cluster (Fig. 2B). This suggests that identification of the query cluster could lead to selection of lower-RMSD templates.

To improve the accuracy with which we identify query sequences’ CDR clusters, we first sought to understand why the accuracy of CDR cluster identification varies across loop lengths and types. We found that accuracy is affected by (1) the number of clusters in each loop length and type, (2) the number of loops populating each cluster, and (3) the total number of loops of a given length and type (Fig. S1). First, we found that loops with a larger number of clusters tended to have lower accuracy. For example, H1-13 has eight clusters and a blindBLAST assignment accuracy of 78.6 ± 0.4% whereas H2-9 has three clusters and an accuracy of 91.2 ± 0.5%. Second, we found that loops with uniform populations among clusters had lower accuracy. For example, H2-10 and H1-13 both have eight clusters, so based on our first observation we expected their accuracy to be similar. It is not: H2-10 has an accuracy of 73.3 ± 0.1% whereas H1-13 has an accuracy of 78.6 ± 0.4%. Analyzing the populations of the clusters for each loop, we observed that clusters H2-10-1 and H2-10-2 have a similar number of CDRs whereas clusters H1-13-1 have many more CDRs than any other H1-13 cluster (Fig. 1). Third, accuracy can be limited by sparse data. We have observed lower accuracies for loops with a small number of structures for a length and type. This is exemplified by H1-14, L1-12, L3-8, L3-10 having the worst accuracies among all loops: 45.1 ± 0.1%, 72.9 ± 4.8%, 65.4 ± 3.7%, 62.0 ± 2.8% (with a total number of 30, 43, 62, 72 loops), respectively.

**Figure 3:**
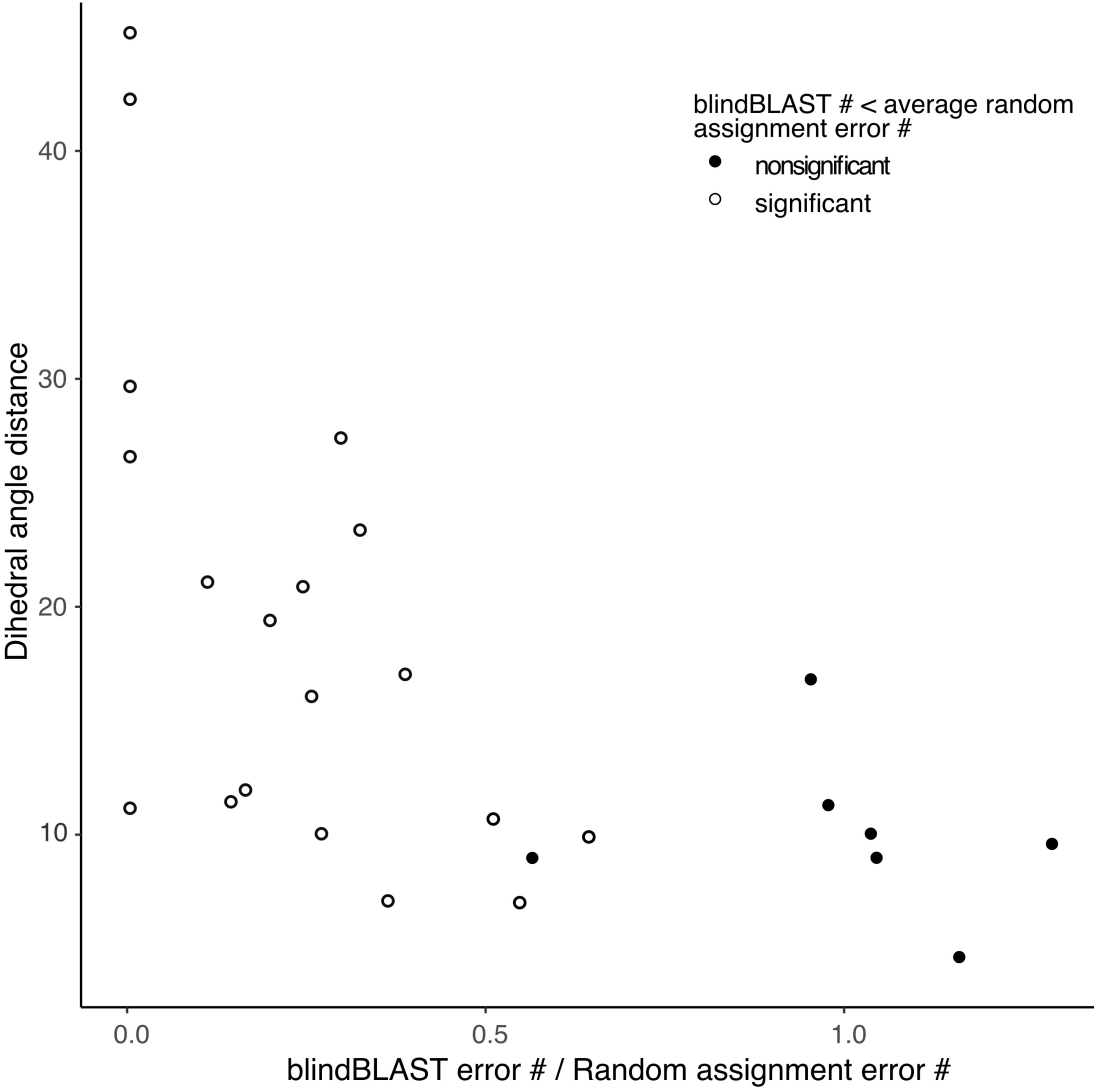
Small distance between a pair of clusters is associated with higher likelihood of misclassification using blindBLAST. Each point corresponds to a misclassification from cluster X → cluster Y, with a minimum of three misclassifications required. The *x* axis is the ratio of the average error count for blindBLAST to the average error count for random assignment. The *y* axis is the dihedral angle distance between the two clusters’ exemplars. BlindBLAST misclassifications are significantly fewer than random when dihedral angle distance between exemplars is large.

In addition, the *χ*^2^ goodness-of-fit test suggests that blindBLAST may classify some loop and length types no better than a random model (Table S2). The sparse loop and length types H1-14, H1-15, H2-9, H2-12, L1-10, L1-15, L1-16, L1-17 are included. The more populated loop and length types including H1-13, H2-10, L2-8 and L3-9 have significantly “better than random” predictions results. Furthermore, errors in each loop and length type consist of several different misclassifications. From the random assignment test, we identified different misclassifications where blindBLAST performs better or worse than random (Table S3 , Fig. S2). The “random-like” misclassifications typically have smaller distances between the true and incorrectly predicted cluster than those of the “better than random” misclassifications (Fig. 3).

**Figure 4:**
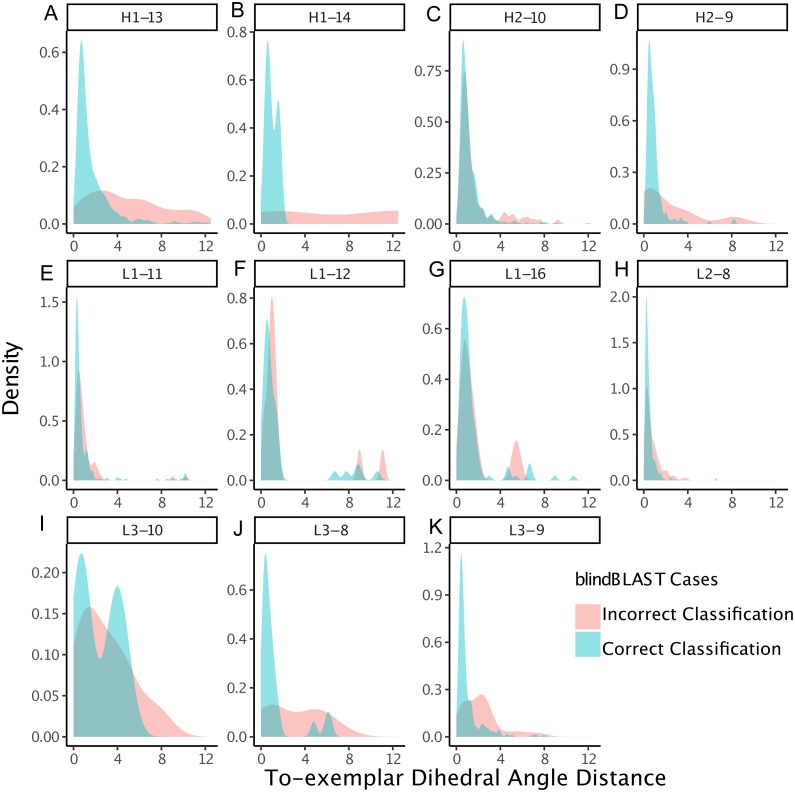
CDRs that are misclassified have a relatively large dihedral angle distance to their cluster exemplars. Density of dihedral angle distances between the query loops and their cluster exemplar loops. For most loop lengths and types (e.g., H1-13, H2-9, L1-15, L3-8, L3-9, and L3-10), the misclassified distribution has more density at larger dihedral angle distances with respect to the correctly classified distribution. The skewedness indicates that for many loops, if a query CDR is distant to its corresponding structural exemplar, then it is more likely to be incorrectly classified using the blindBLAST method.

**Figure 5:**
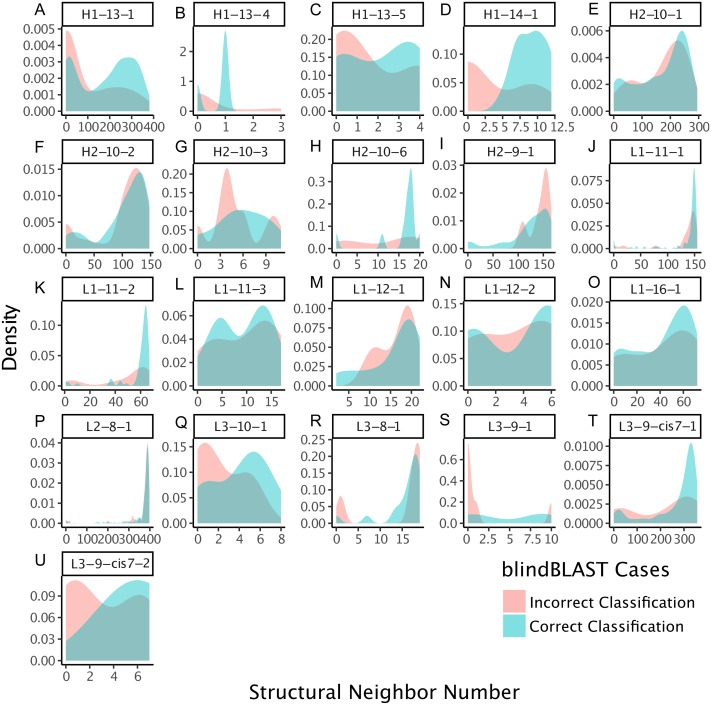
CDRs that are misclassified have fewer structural neighbors, in some clusters. The density of the number of structural neighbors for loop lengths and types with more than five members, both correctly and incorrectly classified. Structural neighbors, as defined in the Methods, are all CDRs with dihedral angle distances equal to or less than 1/15th of the cluster radius to a given CDR. The 1/15th is chosen because it has the best inference. In many clusters, including H1-13-1, H1-13-4, H1-13-5, H1-14-1, H2-10-6, L1-12-1, L3-10-1, L3-9-1, L3-9-cis7-1, L3-9-cis7-2, the misclassified CDRs have greater density at lower numbers of structural neighbors, with respect to the correctly classified CDRs. These data suggest that the number of structural neighbors may affect the chance of correct template selection for a query structure.

We also examined whether where the query CDR is situated inside its cluster affects its chance of being misclassified. We quantify a query CDR inside its cluster by two metrics: (1) the dihedral distance of the query CDR to its cluster exemplar and (2) the number of structural neighbors to the query CDR. The distributions of query–exemplar dihedral-angle distances (Fig. 4) and suggest that query CDRs that are more distant from their corresponding cluster exemplars are more likely to be misclassified by blindBLAST. The distributions of structural neighbor counts (Fig. 5) suggest that for some well populated clusters, such as H1-13-1, H2-9-1 and L3-9-cis7-1, CDRs with fewer neighbors in the same cluster are more likely to be misclassified. Taken together, these data indicate that query CDRs that are located centrally within their cluster—those having a small dihedral distance from the cluster exemplar and many neighbors—are more accurately classified.

### GBM improves cluster identification accuracy over blindBLAST

**Figure 6:**
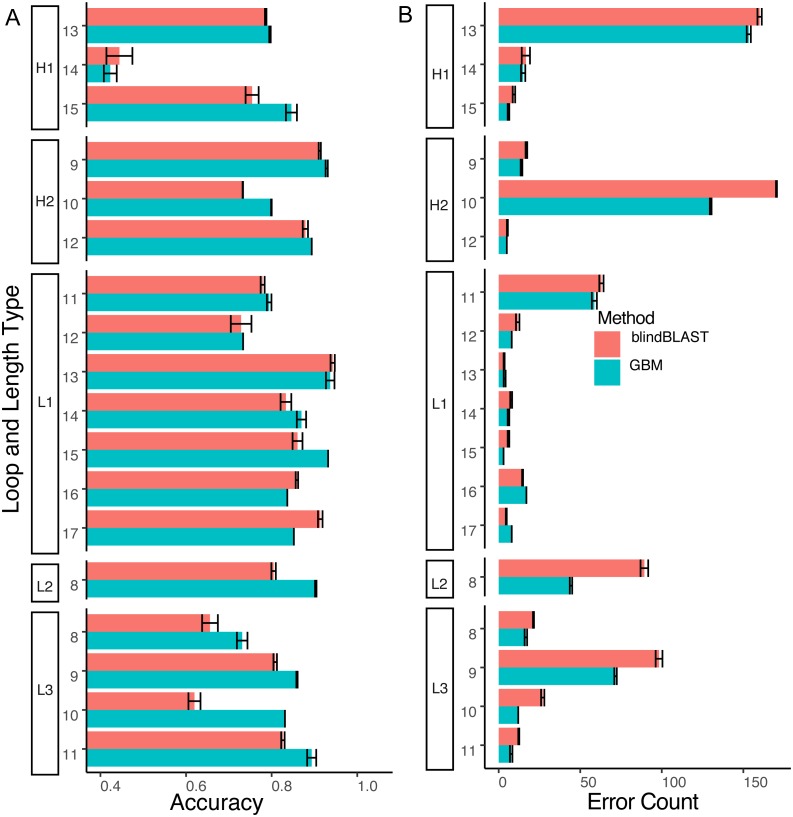
The GBM model has higher accuracy and lower error count than blindBLAST. (A) Comparison of blindBLAST (red) and GBM (blue) accuracy in assigning CDR sequences to clusters. Error counts and accuracies are averaged over each of 3 repeats (in the 3-repeat 10-fold CV scheme) for both GBM and blindBLAST (see Methods). (B) Comparison of the number of erroneously assigned clusters in blindBLAST and GBM error count. The GBM model universally lowers error count.

Compared to blindBLAST, GBM models improve average query cluster identification accuracy from 79.0% ±  0.23% to 83.4% ± 0.11% (Fig. 6). The difference between GBM and blindBLAST accuracy is greater than the standard deviation calculated across each repeat of the three repeats in the 10-fold cross-validation scheme. Also, the GBM model accuracy variance arises from the sparsity of data as evidenced by the larger variance in the loops with sparser members (Fig. S3).

**Figure 7:**
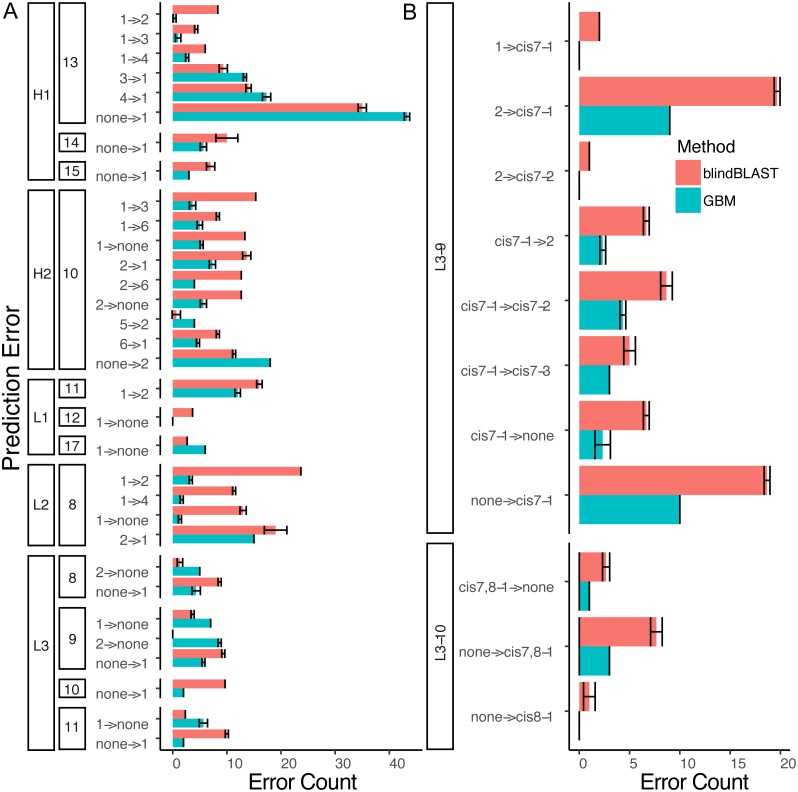
A detailed dissection of error count reduction by the GBM model. (A) All misclassifications with a difference of three or more in error count between GBM and blindBLAST are plotted. A misclassification labeled as 1 →2 denotes queries belonging to cluster 1 that are incorrectly classified as cluster 2. (B) Misclassifications involving at least one cis cluster with corresponding blindBLAST and GBM error counts.

**Figure 8:**
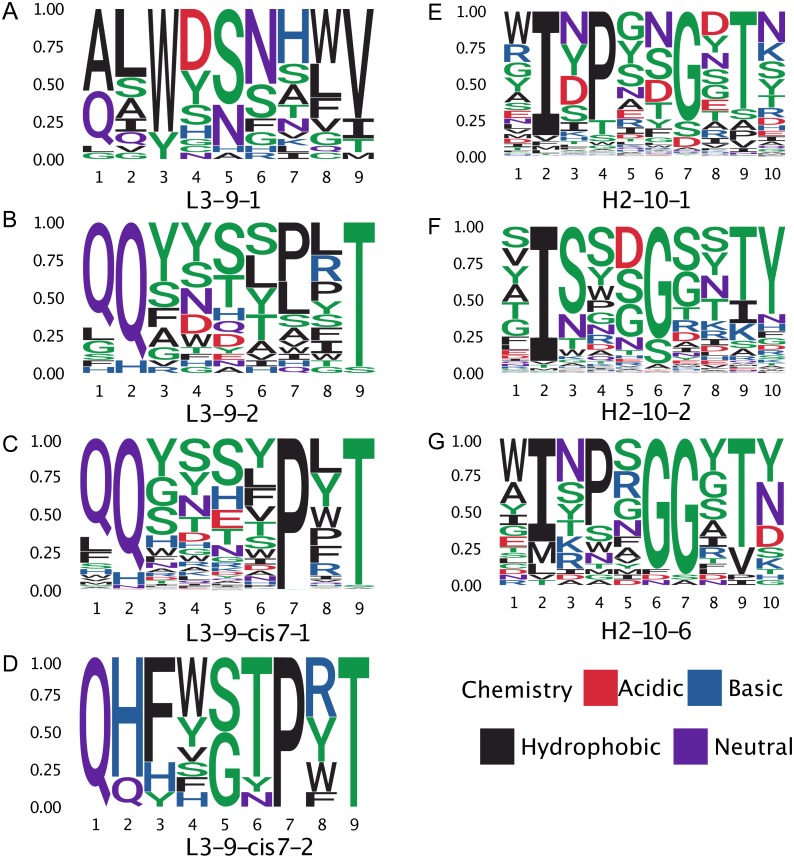
Sequence logos of selected CDR clusters show there are no readily available sequence rules. (A–D) The amino acid compositions of the L3-9 clusters. There are no universal distinguishing residues, except for L3-9-1 which does not contain a proline at position 7. (E–G) Similarly, for H2-10-1, -2, and -6 there is not a universal difference in sequences.

**Figure 9:**
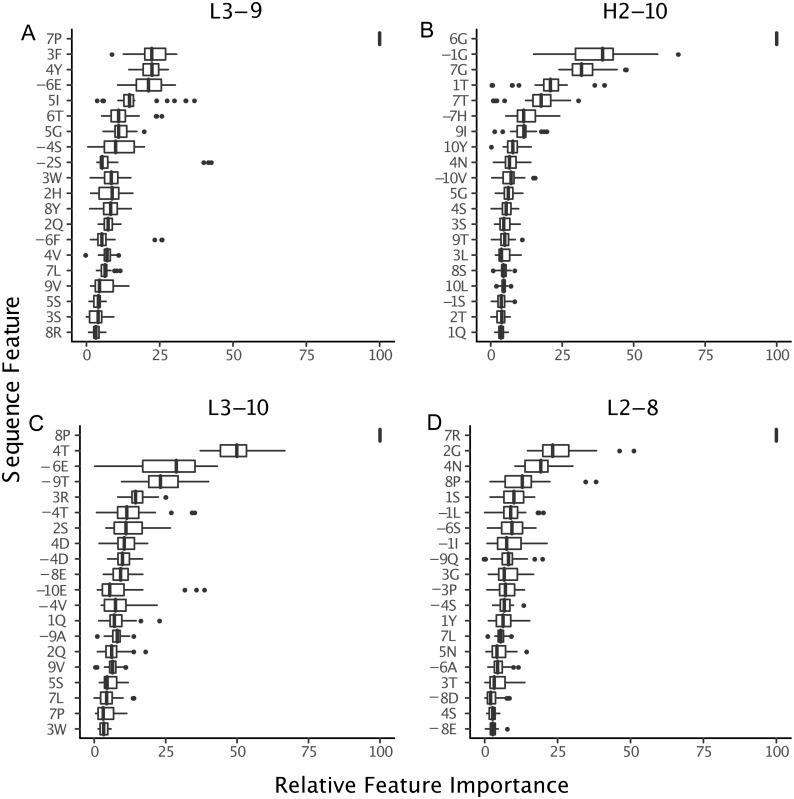
Relative importance can be extracted from GBM models, permitting the identification of key sequence features. For the best GBM model of each loop and length type (though only L3-9 (A), H2-10 (B), L3-10 (C) and L2-8 (D) are shown), the training features were ranked by how much they can help to reduce the training error on a scale of least to most important (1–100). The features are named by the position and the residue type. For example, position “1” is the start of the CDR loop and “−1” is the preceding residue of the loop. Data are from 3-repeat-10-fold splitting between the training/validation and the testing set. As expected for L3-9 and L3-10, two proline containing loops, the presence of a proline at key positions is the most important feature. For H2-10 and L2-8, 6G and 7R are identified as the most important features, respectively. The 6 , 7 and −1 position Gly are the most important features for H2-10 models. In H2-10-6, GBM captured the conserved glycines at positions 6 and 7, which enable the segment to adopt the E region conformation in Ramachandran map. Capturing this feature reduced the error count related to H2-10-6 classification (Fig. 8).

Next, to determine where the improvement in GBM accuracy is achieved, we decomposed the overall error count into changes in individual misclassification counts (Fig. 7) and compared potential sequence rules to key features extracted from the GBM models (Figs. 8 and 9).

Decomposing the error counts into their constituent misclassifications provided a few insights into how GBM models outperform the blindBLAST method. For H2-10 loops, the GBM model improved H2-10-2 → H2-10-X misclassifications over blindBLAST. For example the H2-10-2 → H2-10-1 error count was reduced from 14 to 8 (Fig. 7A). Correspondingly, H2-10-X → H2-10-2 misclassifications increased. For example the H2-10-6 → H2-10-2 error count increased from 4 to 7. Taken together, these observations indicate that blindBLAST was failing to properly classify CDR loops as H2-10-2. Similarly, we examined L1-11 loops, which are similar to H2-10 in that the second cluster is well populated (Fig. 1). Yet, L1-11 loop classification improvements came from fewer L1-11-1 loops being misclassified as L1-11-2, rather than fewer L1-11-2 loops being misclassified as L1-11-X. This case improves less than H2-10, likely because the L1-11-1 and L1-11-2 clusters are already similar, involving just a flip of two residues, while the other dihedrals have conserved structure. Finally, we investigated improvements for L3-9 and L3-10 loops, both loops that occasionally contain *cis* peptide bonds (Fig. 7B). For both loops, we observed that the most drastic improvements came from cases where blindBLAST was incorrectly assigning loops, some without prolines, to *cis* clusters; To BLAST, the penalty of misaligning a proline at the *cis* position is more or less equal to the penalty of misaligning any other position, but such parity is not required by a GBM model, which can assign greater importance to having a proline in the *cis* position. Other than promoting the importance of the proline residue at a *cis* position using machine learning, another approach is to filter out any template from a cis-cluster. The GBM method beats the filtering method in identifying cis-related clusters in several situations. It reduced prediction errors involving query sequences with cis-proline residue and a non-proline bearing template candidate. It also reduced errors in which query sequences from one *cis*-proline cluster incorrectly identified as some other *cis*-proline cluster (cis7-1 →cis7-2). In some other cases, reduced errors are those which cis-proline loops are predicted into clusters with non-cis-proline (cis7-1 →2 with nine cases in L3-9-2 having 7th-non-cis-proline, cis7-1 →. None with ten 7th-position-non-cis-proline cases in L3-9-none) which the incorrectly predicted cases can’t be excluded by proline filtering in the 7th position.

The proline observation raised the question: were there any other sequence features missed by BLAST, but identified by GBM models? To this end, we constructed sequence logos (Crooks et al., 2004) of select CDR loop clusters (Fig. 8) and compared them to residue features deemed important in our GBM models (Fig. 9). For L3-9, we observed proline at position 7 in the loop to be key in both *cis* clusters and the most important feature for our GBM model. The GBM model additionally identified a key threonine residue at position 6, which is never present in L3-9-1. Results are similar for L3-10, with proline residues at *cis* positions being by far the most important. For H2-10, the most important feature of glycine at the 6 position, and the second and third important features are glycine at the −1 and 7 positions. The accuracy improvements arise not from a single residue presence or absence but from the combination of many features with varying weights in the training process.

### Comparison to other methods

Several other groups have attempted to predict CDR canonical cluster membership. Two other comparable methods are PIGS and SCALOP. SCALOP (Wong et al., 2018) uses PSSMs derived for the length-independent clusters identified first by Nowak et al. (2016). They then assign membership to the highest scoring cluster based on the PSSMs, except for the L2 CDR which is always assigned to the most populous cluster. On the other hand, PIGS (Chailyan et al., 2011; Marcatili et al., 2014) classifies based on residue identities at key positions of *γ* light chain CDR clusters and heavy chain clusters curated from previous literature, as well as using clusters found by agglomerative clustering on *λ* light chain CDR loops using TM-score distance as distance function. In addition to using different cluster definitions, both methods self-report measures of accuracy that differ from our own, confounding direct comparisons with our work.

SCALOP reports precision or the number of predictions identifying a cluster with a loop within 1.5 Å backbone RMSD to the target, dived by the total number of predictions. They report precisions of 89.26% for the CDR H1 loop, 93.60% (H2), 95.67% (L1), 99.13% (L2), and 93.31% (L3). We can compute a similar measure, albeit for different clusters: the number of correct cluster predictions divided by the number of total cluster predictions. The GBM achieves precisions of 85.65% (H1), 88.13% (H2), 87.67% (L1), 93.15% (L2), 87.94% (L3) without accounting for the “none” clusters, same as how SCALOP reports the precisions. These values are unsurprisingly lower as we attempt to classify into greater number of possible clusters.

In their recent update to the webserver PIGSPro (Lepore et al., 2017), Lepore et al. report only the average C*α* RMSD of all loops (including CDR H3) following alignment of framework regions. They report an average value of 1.79 ±  1.03 Å. Our GBM achieves 1.03 ±  1.06 Å in a comparison of the CDRs, excluding the H3, identified by our GBM-improved BLAST. As expected, our reported average RMSD is lower because we are not considering the difficult-to-model CDR-H3 loop.

## Discussion

In the current implementation of RosettaAntibody, blindBLAST is used to select templates for the non-H3 CDR loops, so we are interested in investigating and improving cases where blindBLAST identifies templates with high RMSD. We found that one way to improve template RMSD is to take advantage of the fact that non-H3 CDR loops cluster and to search for templates within the query cluster. BlindBLAST does not consider cluster information explicitly, instead it selects templates of the same length and loop type based on sequence similarity alone. When we tested the ability of blindBLAST to identify clusters compared to a random model, we found multiple and diverse sources of error in cluster classification, so we turned to machine learning, and in particular a GBM model, to improve classification accuracy.

When comparing blindBLAST to a random model, we observed various potential sources, but found no single cause, for inaccurate cluster assignment. First, some loops with few structures, such as H1-14, H1-15, L3-8 were more difficult to classify than loops with many structures because many of the associated misclassifications from the blindBLAST result have random-like error counts (Table S3). Second, other loops with many unbalanced clusters (i.e., where most loops belong to one cluster and few loops belong to the remaining clusters), such as H1-13, resulted in low accuracy. For these loops, identifying a low-RMSD template is confounded by the sparsity of potential templates in the cognate cluster and the large number of potential templates in the other clusters (Fig. S1). Assuming a high sequence similarity across all loops of the same length and type, it is likely that the highest bitscore will arise from an alignment to the most popular cluster. Indeed, we observe misclassifications from sparsely populated clusters to the most populated cluster frequently (6/15 times, Table S4) when blindBLAST performs worse than random. Third, low accuracy was observed when there were two clusters with approximately equal membership. H2-10 loops, which have two highly populated clusters (1 and 2), are such an example and account for 2 of the 15 misclassifications when blindBLAST performs worse than random. Additionally, blindBLAST misclassifies loops where clusters have small dihedral-angle distance between exemplars, such as between L2-8-1 and L2-8-2 (4.5) and between L2-8-1 and L2-8-4 (8.8). Furthermore, in many loop and length types, queries lying at a greater dihedral-angle distance to the cluster exemplar and with a smaller number of structural neighbors were found to have a greater chance to be misclassified.

With no single clear source for blindBLAST misclassifications, we turned to GBM models to improve classification accuracy. As shown in Fig. 7, GBM can better distinguish some cluster pairs with even relatively small amounts of structural data. For example, misclassifications from L3-9-2 to L3-9-cis7-1, from H2-10-1 to H2-10-6, and from L2-8-1 to L2-8-4 have reduced error counts despite small dihedral-angle distances between their cluster exemplars (6.9, 6.8 and 8.8, respectively). However, better performance was not observable for misclassifications involving clusters L2-8-1 and L2-8-2 with only 4.45 dihedral-angle distance between exemplars.

For clusters with relatively large between-clusters-dihedral-angle-distance, GBM models may still not offer any improvement, such as the misclassification between cluster pairs H1-13-4 & H1-13-1 or H1-13-6 & H1-13-1 with dihedral-angle distances of 17 and 23 between their exemplars, respectively. Having the lack of improvements for such cases in mind, along with the fact that most misclassifications with reduced error counts with GBM models involve clusters that have relatively abundant sample number, we propose that the abundance of data in the non-dominant clusters of the cluster pairs affects how effectively GBM models can improve the blindBLAST performance.

Overall, our results suggest that relative to blindBLAST, GBM is able to better capture features and assign more sensible feature importance with only limited data. GBM models test results have reduced error count (>3) in nine out of 15 listed blindBLAST “worse than random” misclassifications, in 14 out of 33 listed blindBLAST “random-like” misclassifications, and 12 out of 21 listed blindBLAST “better than random” misclassifications.

## Conclusions

In summary, our study has demonstrated that a CDR template from the corresponding structural cluster generally has lower RMSD than a template from the wrong cluster. We have examined the ability of blindBLAST, which is the method used by RosettaAntibody, to identify non-H3 CDR loop clusters implicitly. We trained a GBM model for each CDR loop and length type, and cumulatively improved the canonical structural cluster identification accuracy from 79.0% (±0.23%) test accuracy using the blindBLAST approach in RosettaAntibody to 83.4% ±  0.11% test accuracy using GBM models. If we remove the query cases from the “none” clusters because predicting a loop correctly as a “none” cluster may not narrow the template candidates, then the test accuracy improves from 84.5% ± 0.24% for blindBLAST to 88.16% ± 0.056% for the GBM. The GBM model reduces error counts in all categories of misclassification we benchmarked for blindBLAST. However, most of the misclassifications with GBM reduced error counts involve clusters with relatively abundant sample sizes, especially the non-dominant clusters. Thus, the bottlenecks to further improvement are primarily the member size imbalance between clusters and data sparsity in clusters. Methods that can generate valid data to enrich clusters with sparse data may improve the estimation accuracy of the GBM model. A set of structures that lie within the cluster radius constraint could be generated using Rosetta, emulating the SMOTE method (Chawla et al., 2002) for enriching samples in underpopulated classes. Another approach that serves to increase the member sizes of these clusters is to use semi-unsupervised learning to incorporate the sequenced antibodies without solved structures.

Furthermore, the GBM models are found incapable of further reducing errors in misclassifications involving clusters with small dihedral angle distance such as between L2-8-1 and L2-8-2. To address this limitation, we may wish to reflect the differences of distances between cluster pairs in the loss function in the machine learning training process using generated synthetic data, so that mismatches between clusters of greater structural differences can be penalized more heavily. On the other hand, the sampling and learning process can also be adjusted by training each weak learner with an under-sampled dominant cluster rather than oversampling the non-dominant clusters used in this study. Finally, instead of ten residues upstream and downstream of the loop proper used in our method, antibody framework residues which are neighboring the CDR loop residues are known to affect loop conformation and could also be included as features (Ting et al., 2010).

##  Supplemental Information

10.7717/peerj.6179/supp-1 Figure S1 BlindBLAST accuracy for each loop and length type plotted against secondary features (A) Accuracy versus the total number of cluster members reveals that smaller clusters are harder to predict. (B) Standard deviation improves (lessens) for larger clusters. (C) Accuracy versus the ratio of sizes of the top two populated clusters shows that loops with a single well-populated cluster can be classified with higher accuracy. (D) Accuracy versus number of clusters shows that classifying into small numbers of clusters can be low accuracy.

10.7717/peerj.6179/supp-2 Figure S2 BlindBLAST misclassifications can be categorized based on comparison to random assignment (i.e., better, the same, or worse than random) The significance value, as defined in Equation 2, is used to identify if a blindBLAST cluster misclassification is random-like. For each point representing a misclassification , the average error count from random assignment iterations is plotted against the blindBLAST error. A majority of misclassifications have better than random error counts but some misclassifications are identified as worse than random.

10.7717/peerj.6179/supp-3 Figure S3 The optimal hyper-parameters for the GBM models differ from loop type to loop type **Grid-search results for a single fold of the 10 outer cross-validation folds. Each point corresponds to the accuracy ( *y*-axis) averaged over each of the inner 10-fold CV runs using the 9-folds of data. The error bars show the standard deviations across inner folds averaged over the same runs. The horizontal line presents the accuracy of the blindBLAST approach. The *x*-axis captures the number of decision trees (# trees) and the point/line color represents the single weak learner complexity as the number of branches**. In general, as the number of decision trees and the number of branches increases, the models achieve greater accuracy than blindBLAST, though there is no consistent trend. Compared to the performance of blindBLAST, the best model achieves higher mean accuracy.

10.7717/peerj.6179/supp-4 Table S1 Categorization criteria of misclassification based on significance test using random assignment error counts as the null model The misclassifications observed in the blindBLAST results can be divided into three categories, based on p-value (determined against a random assignment simulation, **Equation 2**).

10.7717/peerj.6179/supp-5 Table S2 Loop and length types with sparse CDR data are more likely to have “random-like” blindBLAST prediction result Loops with populated members have significant *χ*^2^ result, indicating the blindBLAST classifies better than randomly. The *p*-values are derived from the *χ*^2^ distribution using the *χ*^2^ as determined by **Equation 1**.

10.7717/peerj.6179/supp-6 Table S3 Confusion matrices. Misclassification types by blindBLAST performance group The number in the confusion matrix is the value of blindBLAST error count subtracting the average random simulation error count. A negative number indicates fewer misclassification errors by blindBLAST when compared to the average random assignment error. Bold indicates that the misclassification error count is significantly different from random. The p values are derived from **Equation 2**.

10.7717/peerj.6179/supp-7 Table S4 Sparse cluster error comparisons between GBM and blindBLAST The error counts for blindBLAST and GBM of misclassifications in sparse query clusters (fewer than 50 CDR loops). Of the 31 sparse query clusters, GBM reduces errors for 16.

## Additional Information and Declarations

### Data Availability

The following information was supplied regarding data availability: The code is available on Github in the xlong2/machine-learning-cdr repository: https://github.com/xlong2/machine-learning-cdr.
