# Supplementary figures and images for: Non-H3 CDR template selection in antibody modeling through machine learning
Source: PeerJ. 2019 Jan 11;7:e6179. doi: 10.7717/peerj.6179 (PMC6330961; doi:10.7717/peerj.6179)

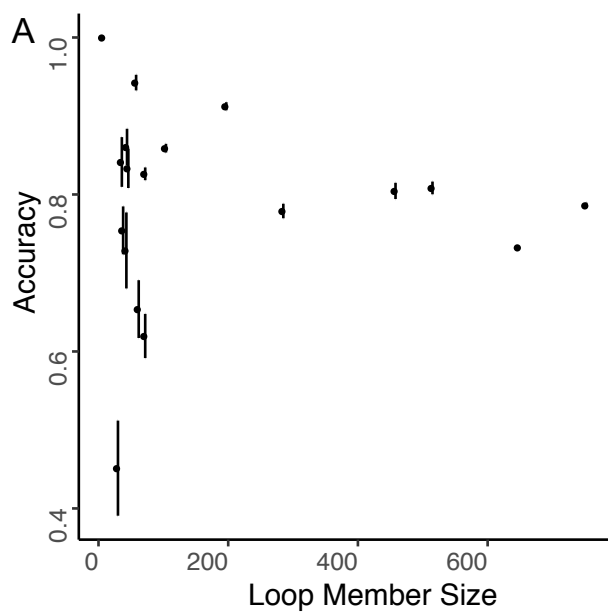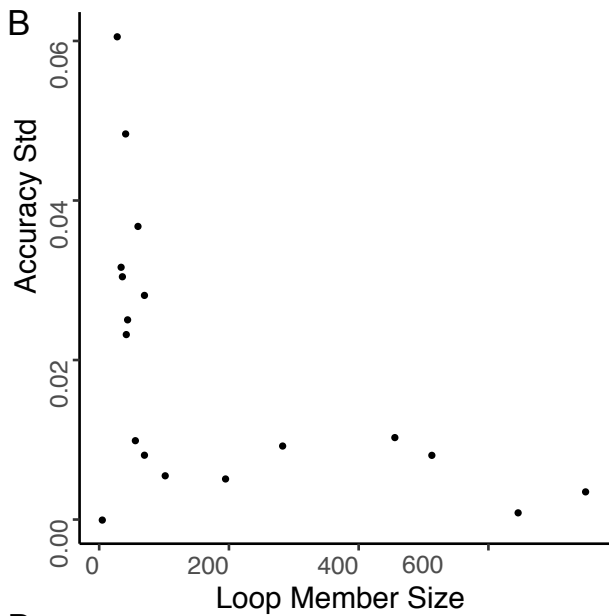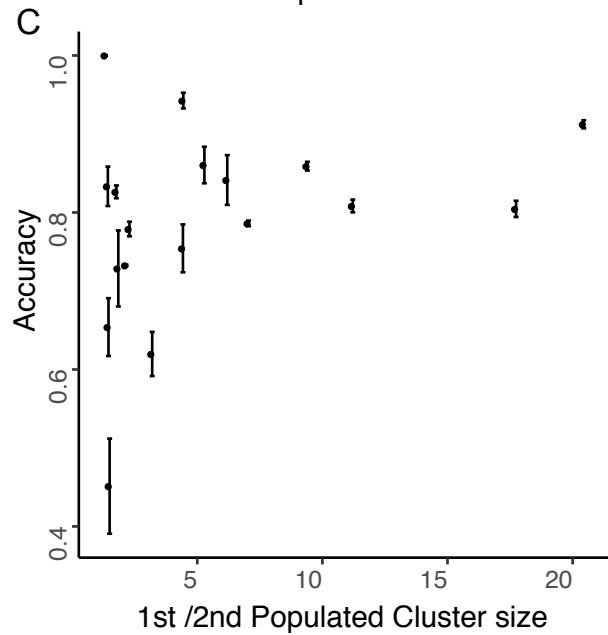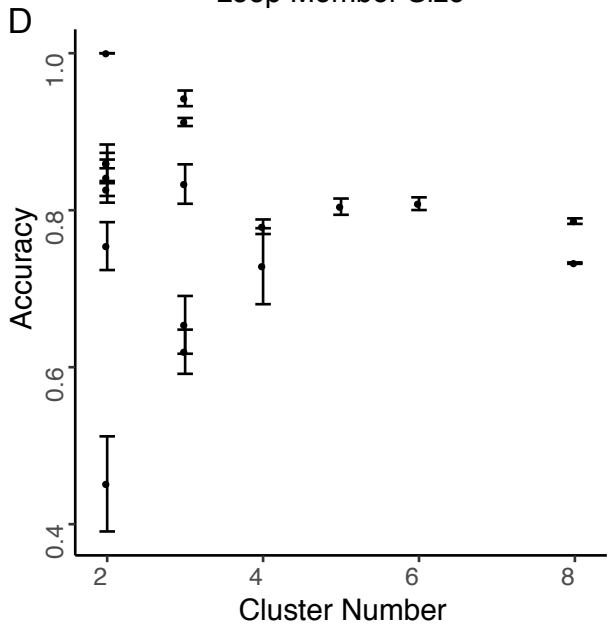

Supplement: Figure S1 — (A) Accuracy versus the total number of cluster members reveals that smaller clusters are harder to predict. (B) Standard deviation improves (lessens) for larger clusters. (C) Accuracy versus the ratio of sizes of the top two populated clusters shows that loops with a single well-populated cluster can be classified with higher accuracy. (D) Accuracy versus number of clusters shows that classifying into small numbers of clusters can be low accuracy. [file peerj-07-6179-s001.pdf]

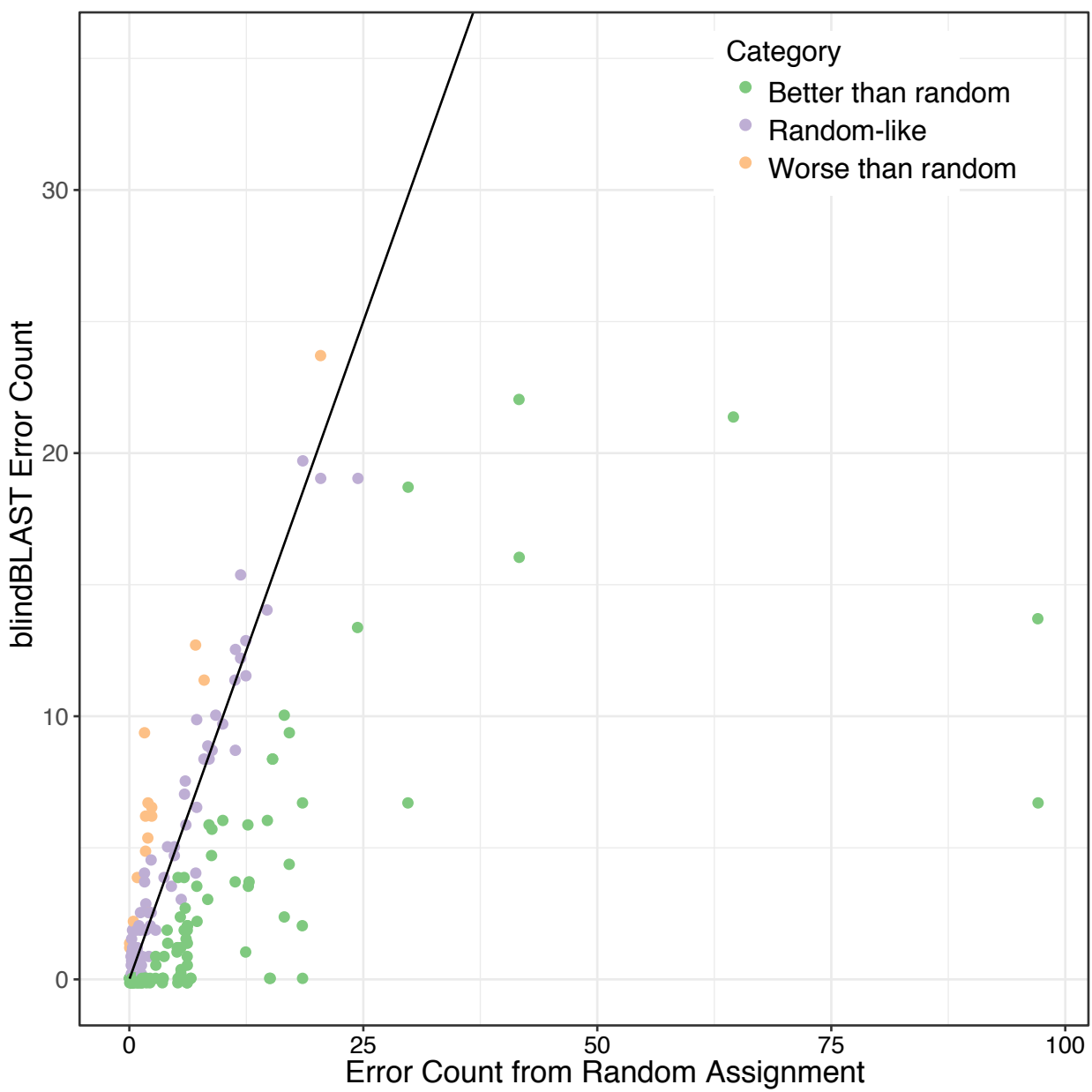

Supplement: Figure S2 — The significance value, as defined in Equation 2, is used to identify if a blindBLAST cluster misclassification is random-like. For each point representing a misclassification , the average error count from random assignment iterations is plotted against the blindBLAST error. A majority of misclassifications have better than random error counts but some misclassifications are identified as worse than random. [file peerj-07-6179-s002.pdf]

Validation Accuracy

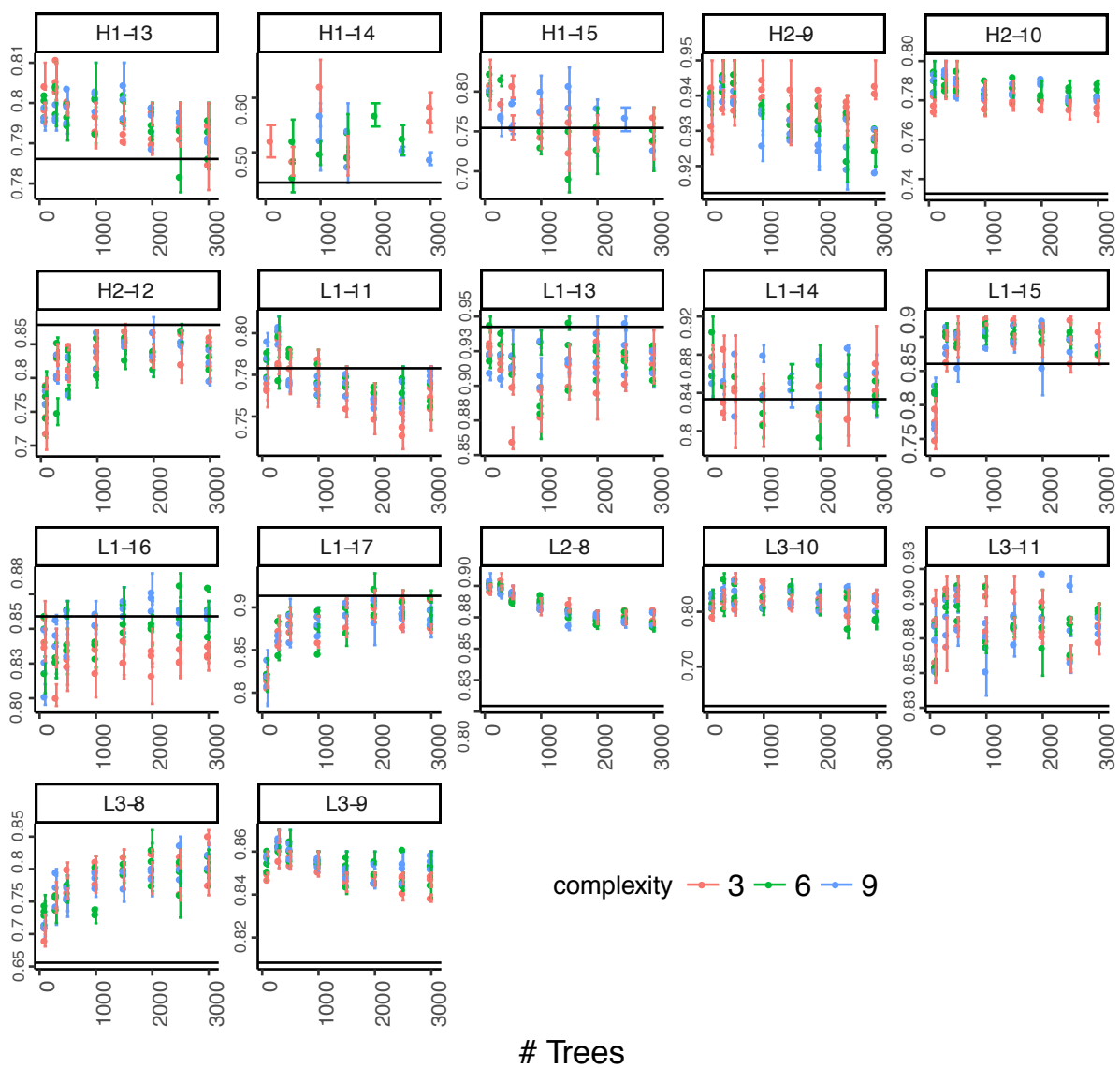

Supplement: Figure S3 — Grid-search results for a single fold of the 10 outer cross-validation folds. Each point corresponds to the accuracy ( y-axis) averaged over each of the inner 10-fold CV runs using the 9-folds of data. The error bars show the standard deviations across inner folds averaged over the same runs. The horizontal line presents the accuracy of the blindBLAST approach. The x-axis captures the number of decision trees (# trees) and the point/line color represents the single weak learner complexity as the number of branches. In general, as the number of decision trees and the number of branches increases, the models achieve greater accuracy than blindBLAST, though there is no consistent trend. Compared to the performance of blindBLAST, the best model achieves higher mean accuracy. [file peerj-07-6179-s003.pdf]
