# Supplementary material for: Non-H3 CDR template selection in antibody modeling through machine learning
Source: PeerJ. 2019 Jan 11;7:e6179. doi: 10.7717/peerj.6179 (PMC6330961; doi:10.7717/peerj.6179)
Supplement: Table S1 — The misclassifications observed in the blindBLAST results can be divided into three categories, based on p-value (determined against a random assignment simulation, Equation 2). [file peerj-07-6179-s004.docx]

| Misclassification Category | p-value |
| --- | --- |
| Worse than random | p > 0.975 |
| Better than random | p < 0.025 |
| Random-like accuracy | 0.025 < p < 0.975 |
