# Supplementary material for: Non-H3 CDR template selection in antibody modeling through machine learning
Source: PeerJ. 2019 Jan 11;7:e6179. doi: 10.7717/peerj.6179 (PMC6330961; doi:10.7717/peerj.6179)
Supplement: Table S2 — Loops with populated members have significant χ2 result, indicating the blindBLAST classifies better than randomly. The p-values are derived from the χ2 distribution using the χ2 as determined by Equation 1. [file peerj-07-6179-s005.docx]

| **CDR** | **P-value** |
| --- | --- |
| H1-13 | 4.73E-178 |
| H1-14 | 0.0950 |
| H1-15 | 0.0447 |
| H2-9 | 0.161 |
| H2-10 | 7.48E-101 |
| H2-12 | 0.0404 |
| L1-10 | 0.0513 |
| L1-11 | 9.60E-67 |
| L1-12 | 2.76E-10 |
| L1-13 | 1.19E-18 |
| L1-14 | 2.84E-12 |
| L1-15 | 0.0368 |
| L1-16 | 0.0651 |
| L1-17 | 0.541 |
| L2-8 | 1.48E-90 |
| L2-12 | 0.00815 |
| L3-8 | 4.07E-07 |
| L3-9 | 1.62E-93 |
| L3-10 | 9.49E-09 |
| L3-11 | 4.29E-07 |
