# Supplementary material for: Non-H3 CDR template selection in antibody modeling through machine learning
Source: PeerJ. 2019 Jan 11;7:e6179. doi: 10.7717/peerj.6179 (PMC6330961; doi:10.7717/peerj.6179)
Supplement: Table S3 — The number in the confusion matrix is the value of blindBLAST error count subtracting the average random simulation error count. A negative number indicates fewer misclassification errors by blindBLAST when compared to the average random assignment error. Bold indicates that the misclassification error count is significantly different from random. The p values are derived from Equation 2. [file peerj-07-6179-s006.docx]

|  | H1-13-1 | H1-13-2 | H1-13-3 | H1-13-4 | H1-13-5 | H1-13-6 | H1-13-7 | H1-13-none |
| --- | --- | --- | --- | --- | --- | --- | --- | --- |
| H1-13-1 | **84** | -0.2 | **-12.8** | **-8.8** | **-11.4** | **-4.2** | **-4.2** | **-43.3** |
| H1-13-2 | **-2.6** | 0.8 | 0.7 | 0.1 | **-0.2** | **-0.1** | **-0.1** | 1.5 |
| H1-13-3 | **-7.8** | 0.7 | **1.7** | **-0.6** | **1.9** | 0.4 | **-0.2** | **3.9** |
| H1-13-4 | -0.8 | **-0.3** | **-0.6** | **3.5** | **-0.4** | **-0.2** | **-0.2** | -1.1 |
| H1-13-5 | **-8.8** | **-0.2** | 0.5 | **-0.4** | **6** | **-0.2** | **-0.2** | **3.3** |
| H1-13-6 | **-5.5** | **-0.1** | 0.1 | **-0.2** | **-0.2** | **2.9** | **-0.1** | **3.1** |
| H1-13-7 | **-5.2** | **-0.1** | **-0.2** | **-0.2** | **-0.2** | **-0.1** | **6.9** | **-0.9** |
| H1-13-none | **-29.6** | 0.8 | **4.2** | 0.6 | **4.6** | 0.1 | 0.1 | **16.2** |

|  | H1-14-1 | H1-14-none |
| --- | --- | --- |
| H1-14-1 | 0.5 | -0.5 |
| H1-14-none | 2.8 | **-2.8** |

|  | H1-15-1 | H1-15-none |
| --- | --- | --- |
| H1-15-1 | **3.4** | **-3.4** |
| H1-15-none | 1.3 | **-1.3** |

|  | H2-9-1 | H2-9-3 | H2-9-none |
| --- | --- | --- | --- |
| H2-9-1 | **5.1** | **-2.7** | **-5.4** |
| H2-9-3 | 0.3 | **-0.1** | **-0.2** |
| H2-9-none | 0.6 | **-0.2** | **-0.4** |

|  | H2-10-1 | H2-10-2 | H2-10-3 | H2-10-4 | H2-10-5 | H2-10-6 | H2-10-none |
| --- | --- | --- | --- | --- | --- | --- | --- |
| H2-10-1 | **109** | **-90.4** | 3.4 | **-3.8** | **-2.8** | **-7** | **-11.1** |
| H2-10-2 | **-83.4** | **78.6** | **-4.2** | 0.3 | -1 | **5.5** | 1.3 |
| H2-10-3 | 0.4 | **-5.2** | **6.3** | **-0.3** | **-0.2** | 0.5 | **-1.4** |
| H2-10-4 | **-4.1** | 2.3 | **-0.3** | 0.5 | **1.3** | 1 | **-0.6** |
| H2-10-5 | **-1.8** | -0.7 | **-0.2** | **1.3** | **0** | 0.8 | 0.7 |
| H2-10-6 | **-7** | -3.1 | **-0.9** | 1.6 | **-0.2** | **9.5** | 0.2 |
| H2-10-7 | -0.3 | 0.9 | **-0.1** | **-0.1** | **0** | **-0.2** | **-0.3** |
| H2-10-none | -5.4 | 0 | -0.4 | **-0.6** | **-0.3** | 1.2 | **3.8** |

|  | L1-10-1 | L1-10-none |
| --- | --- | --- |
| L1-10-1 | 0.7 | -0.7 |
| L1-10-none | **-2.3** | 1.3 |

|  | L1-11-1 | L1-11-2 | L1-11-3 | L1-11-none |
| --- | --- | --- | --- | --- |
| L1-11-1 | **49.8** | **-25.7** | **-15.1** | **-9.1** |
| L1-11-2 | **-19.7** | **31.5** | **-6.6** | **-5.2** |
| L1-11-3 | **-15.1** | **-6.5** | **18.3** | **3.3** |
| L1-11-none | **-6.7** | -2.6 | **4.7** | **4.6** |

|  | L1-12-1 | L1-12-2 | L1-12-3 |
| --- | --- | --- | --- |
| L1-12-1 | **4.6** | **-4.9** | **-2.2** |
| L1-12-2 | **-3.6** | **5.4** | **-1.2** |
| L1-12-3 | **-2.2** | **-1.2** | **3.6** |

|  | L1-13-1 | L1-13-2 | L1-13-none |
| --- | --- | --- | --- |
| L1-13-1 | **11.1** | **-6.2** | **-4.9** |
| L1-13-2 | **-6.2** | **7.6** | **-1.4** |
| L1-13-none | **-4.2** | **-1.4** | **5.6** |

|  | L1-14-1 | L1-14-2 | L1-14-none |
| --- | --- | --- | --- |
| L1-14-1 | **8.9** | **-5.2** | **-3.7** |
| L1-14-2 | **-5.2** | **9.6** | **-4.4** |
| L1-14-none | **-3.7** | -0.1 | **3.8** |

| Var1 | L1-15-1 | L1-15-none |
| --- | --- | --- |
| L1-15-1 | **3.9** | **-3.9** |
| L1-15-none | **-1.9** | 0.9 |

|  | L1-16-1 | L1-16-none |
| --- | --- | --- |
| L1-16-1 | **4.4** | **-4.4** |
| L1-16-none | 0.9 | **-0.9** |

|  | L1-17-1 | L1-17-none |
| --- | --- | --- |
| L1-17-1 | **-0.7** | **0.7** |
| L1-17-none | 0.1 | **-0.1** |

|  | L2-8-1 | L2-8-2 | L2-8-3 | L2-8-4 | L2-8-none |
| --- | --- | --- | --- | --- | --- |
| L2-8-1 | **-3.5** | **3.2** | **-3.6** | **3.3** | 0.5 |
| L2-8-2 | -1.4 | 1.8 | **-0.2** | **-0.5** | **-0.7** |
| L2-8-3 | **-3.6** | **-0.2** | **4.0** | **-0.1** | **-0.1** |
| L2-8-4 | 0.3 | **-0.4** | **-0.1** | **-0.2** | 0.4 |
| L2-8-none | -0.8 | **-0.7** | **-0.1** | **1.4** | 0.2 |

|  | L2-12-2 | L2-12-none |
| --- | --- | --- |
| L2-12-2 | 1.7 | **-1.7** |
| L2-12-none | **-1.7** | 1.7 |

|  | L3-8-1 | L3-8-2 | L3-8-none |
| --- | --- | --- | --- |
| L3-8-1 | **9.9** | **-2.2** | **-7.7** |
| L3-8-2 | -0.9 | **3.6** | **-2.8** |
| L3-8-none | -2.7 | 0.9 | 1.8 |

|  | L3-9-1 | L3-9-2 | L3-9-cis7-1 | L3-9-cis7-2 | L3-9-cis7-3 | L3-9-none |
| --- | --- | --- | --- | --- | --- | --- |
| L3-9-1 | **16.3** | **-1.0** | **-16.5** | **-0.5** | **-0.3** | 2.0 |
| L3-9-2 | -0.4 | 0.6 | 1.1 | 0.5 | **-0.3** | **-1.7** |
| L3-9-cis7-1 | **-18.5** | **-11.9** | **53.6** | -0.2 | 0.2 | **-23.2** |
| L3-9-cis7-2 | **-0.5** | **-0.5** | **-3.2** | **5.1** | **-0.1** | **-0.8** |
| L3-9-cis7-3 | **-0.3** | **-0.3** | -0.1 | **-0.1** | **1.3** | **-0.4** |
| L3-9-none | **7.7** | 2.4 | **-11.2** | 1.2 | **-0.4** | -1.7 |

|  | L3-10-1 | L3-10-cis7,8-1 | L3-10-none |
| --- | --- | --- | --- |
| L3-10-1 | **5.9** | **-1.9** | **-4.0** |
| L3-10-cis7,8-1 | **-1.9** | **5.2** | **-3.3** |
| L3-10-none | -0.3 | 1.7 | -3.3 |

|  | L3-11-1 | L3-11-none |
| --- | --- | --- |
| L3-11-1 | **13.3** | **-14.3** |
| L3-11-none | **-6.6** | **6.6** |
