# Supplementary material for: Non-H3 CDR template selection in antibody modeling through machine learning
Source: PeerJ. 2019 Jan 11;7:e6179. doi: 10.7717/peerj.6179 (PMC6330961; doi:10.7717/peerj.6179)
Supplement: Table S4 — The error counts for blindBLAST and GBM of misclassifications in sparse query clusters (fewer than 50 CDR loops). Of the 31 sparse query clusters, GBM reduces errors for 16. [file peerj-07-6179-s007.docx]

| Query cluster | Template cluster | blindBLAST error | GBM error | GBM improvement |
| --- | --- | --- | --- | --- |
| **H1-13-2** | | | | |
| H1-13-2 | H1-13-none | 2.7 | 2.7 | 0.0 |
| H1-13-2 | H1-13-6 | 0.0 | 0.7 | -0.7 |
| H1-13-2 | H1-13-5 | 0.0 | 1.0 | -1.0 |
| H1-13-2 | H1-13-4 | 0.3 | 2.3 | -2.0 |
| H1-13-2 | H1-13-3 | 1.0 | 4.7 | -3.7 |
| H1-13-2 | H1-13-1 | 6.0 | 10.3 | -4.3 |
|  |  |  |  |  |
| **H1-13-3** | | | | |
| H1-13-3 | H1-13-none | 6.3 | 1.0 | 5.3 |
| H1-13-3 | H1-13-6 | 0.7 | 0.3 | 0.3 |
| H1-13-3 | H1-13-5 | 2.3 | 2.3 | 0.0 |
| H1-13-3 | H1-13-4 | 0.0 | 1.3 | -1.3 |
| H1-13-3 | H1-13-2 | 1.0 | 2.3 | -1.3 |
| H1-13-3 | H1-13-1 | 9.3 | 14.0 | -4.7 |
|  |  |  |  |  |
| **H1-13-4** | | | | |
| H1-13-4 | H1-13-1 | 14.0 | 10.7 | 3.3 |
| H1-13-4 | H1-13-none | 1.0 | 1.0 | 0.0 |
| H1-13-4 | H1-13-7 | 0.0 | 0.7 | -0.7 |
| H1-13-4 | H1-13-3 | 0.0 | 1.0 | -1.0 |
| H1-13-4 | H1-13-2 | 0.0 | 1.3 | -1.3 |
|  |  |  |  |  |
| **H1-13-5** | | | | |
| H1-13-5 | H1-13-none | 5.0 | 0.0 | 5.0 |
| H1-13-5 | H1-13-7 | 0.0 | 0.3 | -0.3 |
| H1-13-5 | H1-13-1 | 3.7 | 4.0 | -0.3 |
| H1-13-5 | H1-13-3 | 1.0 | 2.0 | -1.0 |
| H1-13-5 | H1-13-6 | 0.0 | 1.0 | -1.0 |
| H1-13-6 | H1-13-none | 4.0 | 1.7 | 2.3 |
| H1-13-6 | H1-13-2 | 0.0 | 0.3 | -0.3 |
| H1-13-6 | H1-13-7 | 0.0 | 1.3 | -1.3 |
| H1-13-6 | H1-13-1 | 0.7 | 2.0 | -1.3 |
| H1-13-6 | H1-13-3 | 0.3 | 2.0 | -1.7 |
| H1-13-6 | H1-13-5 | 0.0 | 2.0 | -2.0 |
|  |  |  |  |  |
| **H1-13-7** | | | | |
| H1-13-7 | H1-13-5 | 0.0 | 1.0 | -1.0 |
| H1-13-7 | H1-13-6 | 0.0 | 1.0 | -1.0 |
| H1-13-7 | H1-13-1 | 1.0 | 3.0 | -2.0 |
|  |  |  |  |  |
| **H2-10-3** | | | | |
| H2-10-3 | H2-10-1 | 12.3 | 11.7 | 0.7 |
| H2-10-3 | H2-10-2 | 0.3 | 0.0 | 0.3 |
| H2-10-3 | H2-10-6 | 1.3 | 1.3 | 0.0 |
| H2-10-3 | H2-10-none | 0.0 | 0.7 | -0.7 |
|  |  |  |  |  |
| **H2-10-4** | | | | |
| H2-10-4 | H2-10-6 | 1.3 | 0.3 | 1.0 |
| H2-10-4 | H2-10-1 | 1.0 | 0.7 | 0.3 |
| H2-10-4 | H2-10-5 | 1.3 | 1.0 | 0.3 |
| H2-10-4 | H2-10-2 | 4.7 | 7.0 | -2.3 |
|  |  |  |  |  |
| **H2-10-5** | | | | |
| H2-10-5 | H2-10-4 | 1.3 | 0.0 | 1.3 |
| H2-10-5 | H2-10-6 | 1.0 | 0.0 | 1.0 |
| H2-10-5 | H2-10-none | 1.0 | 0.0 | 1.0 |
| H2-10-5 | H2-10-1 | 1.0 | 1.0 | 0.0 |
| H2-10-5 | H2-10-2 | 0.7 | 4.0 | -3.3 |
|  |  |  |  |  |
| **H2-10-6** | | | | |
| H2-10-6 | H2-10-1 | 8.3 | 5.0 | 3.3 |
| H2-10-6 | H2-10-4 | 2.0 | 0.0 | 2.0 |
| H2-10-6 | H2-10-none | 2.0 | 0.7 | 1.3 |
| H2-10-6 | H2-10-2 | 4.0 | 6.7 | -2.7 |
|  |  |  |  |  |
| **H2-10-7** | | | | |
| H2-10-7 | H2-10-1 | 2.0 | 3.0 | -1.0 |
| H2-10-7 | H2-10-2 | 2.0 | 5.0 | -3.0 |
|  |  |  |  |  |
| **H2-9-3** | | | | |
| H2-9-3 | H2-9-1 | 4.0 | 3.0 | 1.0 |
| H2-9-3 | H2-9-none | 0.0 | 1.0 | -1.0 |
|  |  |  |  |  |
| **L1-11-3** | | | | |
| L1-11-3 | L1-11-none | 5.3 | 3.7 | 1.7 |
| L1-11-3 | L1-11-2 | 0.0 | 0.3 | -0.3 |
| L1-11-3 | L1-11-1 | 0.0 | 1.0 | -1.0 |
|  |  |  |  |  |
| **L1-12-1** | | | | |
| L1-12-1 | L1-12-none | 3.7 | 0.0 | 3.7 |
| L1-12-1 | L1-12-2 | 2.3 | 3.7 | -1.3 |
|  |  |  |  |  |
| **L1-12-2** | | | | |
| L1-12-2 | L1-12-1 | 3.7 | 2.7 | 1.0 |
|  |  |  |  |  |
| **L1-13-1** | | | | |
| L1-13-1 | L1-13-none | 1.3 | 0.0 | 1.3 |
|  |  |  |  |  |
| **L1-14-1** | | | | |
| L1-14-1 | L1-14-2 | 0.0 | 0.7 | -0.7 |
|  |  |  |  |  |
| **L1-14-2** | | | | |
| L1-14-2 | L1-14-none | 1.7 | 0.3 | 1.3 |
| L1-14-2 | L1-14-1 | 0.0 | 1.7 | -1.7 |
|  |  |  |  |  |
| **L1-15-1** | | | | |
| L1-15-1 | L1-15-none | 2.0 | 1.0 | 1.0 |
| **L2-8-2** | | | | |
| L2-8-2 | L2-8-1 | 19.0 | 17.0 | 2.0 |
|  |  |  |  |  |
| **L2-8-3** | | | | |
| L2-8-3 | L2-8-1 | 0.0 | 2.0 | -2.0 |
|  |  |  |  |  |
| **L2-8-4** | | | | |
| L2-8-4 | L2-8-none | 0.7 | 0.0 | 0.7 |
| L2-8-4 | L2-8-1 | 8.3 | 8.3 | 0.0 |
| L2-8-4 | L2-8-2 | 0.0 | 0.3 | -0.3 |
| L2-8-4 | L2-8-3 | 0.0 | 0.3 | -0.3 |
|  |  |  |  |  |
| **L3-10-1** | | | | |
| L3-10-1 | L3-10-none | 6.0 | 2.3 | 3.7 |
|  |  |  |  |  |
| **L3-10-cis7,8-1** | | | | |
| L3-10-cis7,8-1 | L3-10-none | 2.7 | 0.0 | 2.7 |
|  |  |  |  |  |
| **L3-11-1** | | | | |
| L3-11-1 | L3-11-none | 2.3 | 0.7 | 1.7 |
|  |  |  |  |  |
| **L3-8-1** | | | | |
| L3-8-1 | L3-8-none | 5.5 | 2.0 | 3.5 |
| L3-8-1 | L3-8-2 | 1.0 | 4.7 | -3.7 |
|  |  |  |  |  |
| **L3-8-2** | | | | |
| L3-8-2 | L3-8-none | 2.0 | 0.3 | 1.7 |
| L3-8-2 | L3-8-1 | 3.0 | 3.7 | -0.7 |
|  |  |  |  |  |
| **L3-9-1** | | | | |
| L3-9-1 | L3-9-none | 3.7 | 0.0 | 3.7 |
| L3-9-1 | L3-9-cis7-1 | 2.0 | 0.0 | 2.0 |
| L3-9-1 | L3-9-cis7-2 | 0.0 | 2.0 | -2.0 |
| L3-9-1 | L3-9-2 | 0.0 | 5.3 | -5.3 |
|  |  |  |  |  |
| **L3-9-2** | | | | |
| L3-9-2 | L3-9-cis7-1 | 19.7 | 11.3 | 8.3 |
| L3-9-2 | L3-9-cis7-2 | 1.0 | 1.0 | 0.0 |
| L3-9-2 | L3-9-1 | 0.7 | 5.0 | -4.3 |
|  |  |  |  |  |
| **L3-9-cis7-2** | | | | |
| L3-9-cis7-2 | L3-9-cis7-1 | 5.7 | 3.7 | 2.0 |
|  |  |  |  |  |
| **L3-9-cis7-3** | | | | |
| L3-9-cis7-3 | L3-9-cis7-1 | 4.7 | 6.0 | -1.3 |
